# Is moderate hypofractionation accepted as a new standard of care in north america for prostate cancer patients treated with external beam radiotherapy? Survey of genitourinary expert radiation oncologists

**DOI:** 10.1590/S1677-5538.IBJU.2018.0275

**Published:** 2019-04-01

**Authors:** Shearwood McClelland, Kiri A. Sandler, Catherine Degnin, Yiyi Chen, Arthur Y. Hung, Timur Mitin

**Affiliations:** 1 Department of Radiation Oncology, Indiana University School of Medicine, Indianapolis, IN, U.S.A;; 2 Department of Radiation Medicine, Oregon Health and Science University, Portland, OR, U.S.A;; 3 Department of Radiation Oncology, University of California at Los Angeles, Los Angeles, CA, U.S.A;; 4 Biostatistics Shared Resource, Oregon Health and Science University, Portland, OR, U.S.A

**Keywords:** Prostatic Neoplasms, Dose Hypofractionation, Neoplasm Grading

## Abstract

**Introduction:**

Several recent randomized clinical trials have evaluated hypofractionated regimens against conventionally fractionated EBRT and shown similar effectiveness with conflicting toxicity results. The current view regarding hypofractionation compared to conventional EBRT among North American genitourinary experts for management of prostate cancer has not been investigated.

**Materials and Methods:**

A survey was distributed to 88 practicing North American GU physicians serving on decision - making committees of cooperative group research organizations. Questions pertained to opinions regarding the default EBRT dose and fractionation for a hypothetical example of a favorable intermediate - risk prostate cancer (Gleason 3 + 4). Treatment recommendations were correlated with practice patterns using Fisher's exact test.

**Results:**

Forty - two respondents (48%) completed the survey. We excluded from analysis two respondents who selected radical hypofractionation with 5 – 12 fractions as a preferred treatment modality. Among the 40 analyzed respondents, 23 (57.5%) recommend conventional fractionation and 17 (42.5%) recommended moderate hypofractionation. No demographic factors were found to be associated with preference for a fractionation regimen. Support for brachytherapy as a first choice treatment modality for low - risk prostate cancer was borderline significantly associated with support for moderate hypofractionated EBRT treatment modality (p = 0.089).

**Conclusions:**

There is an almost equal split among North American GU expert radiation oncologists regarding the appropriateness to consider moderately hypofractionated EBRT as a new standard of care in management of patients with prostate cancer. Physicians who embrace brachytherapy may be more inclined to support moderate hypofractionated regimen for EBRT. It is unclear whether reports with longer follow-ups will impact this balance, or whether national care and reimbursement policies will drive the clinical decisions. In the day and age of patient - centered care delivery, patients should receive an objective recommendation based on available clinical evidence. The stark division among GU experts may influence the design of future clinical trials utilizing EBRT for patients with prostate cancer.

## INTRODUCTION

The standard eight-to-nine week course of conventional external beam radiation therapy (EBRT) for prostate cancer although effective, disrupts patients’ normal lives, causes financial toxicity to patients and places a significant financial strain on the healthcare system. For these reasons, hypofractionated radiation therapy (RT), which involves larger radiation doses administered over an overall shorter time period, has increased in popularity, and has been established in other disease sites, such as breast cancer, bone metastases, bladder cancer, glioblastoma and non - small cell lung cancer (1-5). Four randomized clinical trials have compared moderately fractionated regimens to conventionally fractionated RT in prostate cancer (Table-1) (6-11). With 5-years of follow-up, none revealed inferiority of hypofractionation regarding the treatment outcomes, and the toxicity reports are contradictory, with no overwhelming and reproducible toxicity associated with a moderately hypofractionated regimens using 2.5 to 3 Gy per fraction. We sought to determine the current view of moderate hypofractionation among North American genitourinary (GU) radiation oncology experts due to their influence in shaping the direction of national guidelines.

**Table 1:** Summary of the four randomized clinical trials comparing hypofractionation (H-RT) with conventional fractionation (C-RT) for prostate cancer (OS = overall survival; DFS = disease-free survival; RFS = relapse-free survival; GU = genitourinary; GI = gastrointestinal; CI = confidence interval).

Trial	Hypofractionation regimen	Follow-up duration	Location	Differences in OS or DFS	Differences in GU toxicity between modalities	Differences in GI toxicity between modalities
RTOG 0415 (7)	2.5 Gy x 28	5 years	USA	No	No (late GU trended toward favoring C-RT: p=0.06)	Yes (late GI: p=0.002 favored C-RT)
CHHiP (8)	3 Gy x 20; 3 Gy x 19	5 years	UK, Ireland, Switzerland, New Zealand	No	No	Yes (acutely favoring C-RT; none by week 18)
PROFIT (6)	3 Gy x 20	5 years	Canada, Australia, France	No	No (acutely; late toxicity favored H-RT)	No (acutely; late toxicity favored H-RT)
HYPRO (9-11)	3.4 Gy x 19	5 years	Netherlands	No	Yes (H-RT inferior for acute and late grade 3+ toxicity)	Yes (H-RT inferior for acute but not late grade 3+ toxicity)

Trial	GU toxicity (H-RT)	GU toxicity (C-RT)	GI toxicity (H-RT)	GI toxicity (C-RT)	Disease control (H-RT)	Disease control (C-RT)
RTOG 0415 (7)	Early grade 2-4 GU = 147/545 Late grade 2-4 GU = 161/545	Early grade 2-4 GU = 145/534 Late grade 2-4 GU = 121/534	Early grade 2-4 GI = 58/545 Late grade 2-4 GI = 121/545	Early grade 2-4 GI = 55/534 Late grade 2-4 GI = 75/534	86.3% DFS (95% CI: 83.1-89.0)	85.3% DFS (95% CI: 81.9-88.1)
CHHiP (8)	Early grade 2-4 GU = 46-49% Late grade 2-4 GU = 6.6-11.7%	Early grade 2-4 GU = 46% Late grade 2-4 GU = 9.1%	Early grade 2-4 GI = 38% Late grade 2-4 GI = 11.3-11.9%	Early grade 2-4 GI = 25% Late grade 2-4 GI = 13.7%	85.9-90.6% biochemical/clinical failure freedom	88.3% biochemical/clinical failure freedom
PROFIT (6)	Early grade 2-4 GU = 185/608 Late grade 2-4 GU = 136/608	Early grade 2-4 GU = 183/598 Late grade 3-4 GU = 134/598	Early grade 2-4 GI = 99/608 Late grade 2-4 GI = 54/608	Early grade 2-4 GI = 62/598 Late grade 2-4 GI = 83/598	85% DFS	85% DFS
HYPRO (9-11)	Early grade 2-4 GU = 75/410 Late grade 2-4 GU at three years = 163/395	Early grade 2-4 GU = 73/410 Late grade 3-4 GU at three years = 151/387	Early grade 2-4 GI = 42/410 Late grade 2-4 GI at three years = 86/395	Early grade 2-4 GI = 43/410 Late grade 2-4 GI at three years = 68/387	80.5% five-year RFS (95% CI: 75.7-84.4)	77.1% five-year RFS (95% CI: 71.9-81.5)

## MATERIALS AND METHODS

### Survey design and deployment

The survey was designed to assess the opinions of GU experts on the default EBRT dose and fractionation for a hypothetical patient with a favorable - intermediate risk prostate cancer who would require by most current conventions EBRT to prostate alone without prophylactic irradiation of pelvic lymph nodes. Three fractionation schemes were offered as choices: conventional fractionation (78 Gy in 2 Gy fractions, 79.2 Gy in 1.8 Gy fractions or equivalent), moderate hypofractionation (70 Gy in 2.5 Gy fractions or equivalent), or SBRT / radical hypofractionation (5 – 12 fractions or equivalent). The study was approved by IRB and electronically sent to 88 North American GU oncology physicians, who serve on cooperative group research organizations such as NRG Oncology. The survey was designed and hosted by Research Electronic Data Capture (REDCap), and contained screening questions to ensure respondents were currently practicing, not in training, and specializing in GU oncology (12). A copy of the survey is available in the Appendix 1.

### Statistical analysis

Based on responses, participants were categorized as “supporters” or “opponents” of moderate hypofractionation. For the purposes of this study, only responders choosing conventional fractionation or moderate hypofractionation were included. Fisher's exact test was used to determine whether treatment recommendations were correlated with practice patterns. R (R version 3.3.3 (2017-03-06)) was used for all data analysis. Statistical significance was set at p < 0.05.

## RESULTS

**Figure 1:**
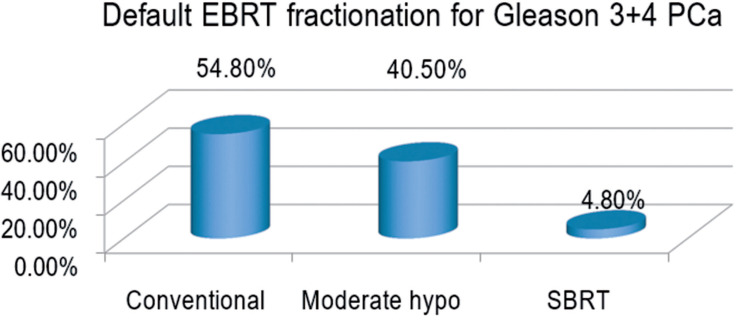
Default External Beam Radiation Therapy Fractionation used by North American genitourinary oncology expert radiation oncologists for treatment of a hypothetical patient with a favorable intermediate risk Prostate Cancer (Gleason 3+4). **PCa** = prostate cancer; **hypo** = hypofractionation

Forty - two of the 88 radiation oncologists completed the survey, of whom 40 (95.2%) recommended either conventional fractionation or moderate hypofractionation; two (4.8%) recommended stereotactic body radiation therapy (SBRT) (Figure-1) and were excluded from the analysis. Of 40 analyzable respondents, 23 (57.5%) recommended conventional fractionation and 17 (42.5%) recommended moderate hypofractionation.

No demographic factors (years in practice, geographic location of residency, geographic location of practice, monthly patient volume, practice type) as well as other clinical positions (active surveillance recommendation preference, brachytherapy boost advocacy, self-identification as an expert brachytherapist, likelihood of considering stereotactic body RT for oligometastatic disease, likelihood of prophylactically irradiating pelvic lymph nodes, support of advanced imaging techniques) were significantly associated with support of moderate hypofractionation. Only the choice of brachytherapy as a preferred treatment option for patients with low - risk prostate cancer approached significance for recommendation of hypofractionation (p = 0.089) (Table-2).

**Table 2:** Association between clinical practice recommendations and choice of default dose/fractionation for Gleason 3+4 prostate adenocarcinoma.

Clinical Scenario	Clinical Practice Recommendation	Conventional Fractionation (78 Gy in 2 Gy fractions, 79.2 Gy in 1.8 Gy fractions, or equivalent)	Moderate Hypofractionation (70 Gy in 2.5 Gy fractions or equivalent)	P value
Active surveillance recommendation for Gleason 6 disease	Yes	21 (91.3%)	17 (100%)	0.546
	No	2 (8.7%)	0 (0%)	
Active surveillance recommendation for Gleason 3+4 disease	Yes	3 (13.0%)	4 (23.5%)	0.607
	No	20 (87.0%)	13 (76.5%)	
SBRT for oligometastatic lesions	Yes	18 (78.3%)	12 (70.6%)	0.837
	No	5 (21.7%)	5 (29.4%)	
Treatment of pelvic lymph nodes in localized high-risk prostate cancer	Rarely	9 (39.1%)	4 (23.5%)	0.377
	Often	14 (60.9%)	13 (76.5%)	
Treatment of high-risk prostate cancer	EBRT+ADT	15 (65.2%)	7 (41.2%)	0.305
	EBRT+ADT+ brachytherapy boost	8 (34.8%)	10 (58.8%)	
Believer in advanced-imaging (Novel ligand-based PET imaging)	Yes	14 (60.9%)	14 (82.4%)	0.137
	No	9 (39.1%)	2 (11.8%)	
First choice for treatment of Gleason 6 disease who desires intervention	Brachytherapy	8 (34.8%)	12 (70.6%)	0.089
	EBRT	5 (21.7%)	1 (5.9%)	
	No preference	10 (43.5%)	4 (23.5%)	

## DISCUSSION

Biological considerations of a markedly lower alpha / beta ratio of prostate cancer in comparison to surrounding normal tissues led researchers to clinical investigation of hypofractionated regimens in management of patients with prostate cancer with EBRT (13). Four large international randomized clinical trials have established non - inferiority of moderate hypofractionation (2.5 – 3 Gy per fraction), with varying toxicity results, some supporting conventional, others hypofractionated regimens, but none reporting overwhelming toxicity within the 5 - years of a follow-up period (Table-1) (6-11).

The degree of acceptance / rejection of treatment modalities in North America is to a significant extent shaped by opinions of leading academic physicians who define and periodically update national treatment guidelines, author consensus statements and shape the future clinical trial protocols. Because of this influence, we sought to determine the acceptance of hypofractionation for prostate cancer among North American GU radiation oncology experts (14).

The results of this study indicate that hypofractionated EBRT, defined as 70 Gy in 2.5 Gy fractions or an equivalent regiment, has made significant inroads among North American GU experts in the treatment of prostate cancer, as more than 40% of experts recommended hypofractionated EBRT as their preferred EBRT treatment modality. Nevertheless, 55% of experts still consider conventionally fractionated EBRT as an unchallenged standard of care. Physicians who embrace a shorter treatment modality (brachytherapy), despite possible increase in acute toxicity - also tend to support hypofractionated EBRT. The relatively even duality regarding conventional versus hypofractionated treatment recommendation for intermediate - risk prostate cancer despite the four randomized trials already published on this topic (6-9) speaks to the issue that randomized trials do not necessarily change the standard of care, particularly in the United States, and a significantly longer follow-up is required; this duality is reflected in the most updated clinically localized prostate cancer guidelines published jointly by the American Urological Association, American Society for Radiation Oncology (ASTRO), and the Society of Urologic Oncology (15, 16). Hypofractionation in breast cancer similarly was adopted in other countries much sooner than in the United States, where ASTRO consensus statements, educational sessions and even direct advertisement to patients regarding hypofractionated options and their non - inferiority, led to final acceptance of hypofractionation as a new standard of care. It is unclear whether reimbursement system in the U.S. is partially responsible for a slower update of shorter treatment courses. Limitations of this study are relatively small sample size, despite an impressive (but still below fifty percent) response rate, inability to capture a full range of options due to multiple choice format, and a lack of granularity in addressing the impact of racial demographic of patients being treated (17). Furthermore, the absence of decade - long toxicity and outcome data comparing conventional versus moderate hypofractionation provides an uncertainty of outcomes beyond the five years of currently published results (6-11).

In conclusion, there is currently a nearly even split between radiation oncology experts in North America recommending conventionally fractionated vs moderately hypofractionated EBRT for patients with prostate cancer, based on dramatically different interpretation of results of 4 randomized clinical trials. Longer follow-up of these trials may impact the balance, while national care and reimbursement policies may influence the accepted standard of care.
